# Nucleocapsid formation and RNA synthesis of Marburg virus is dependent on two coiled coil motifs in the nucleoprotein

**DOI:** 10.1186/1743-422X-4-105

**Published:** 2007-10-24

**Authors:** Andrea DiCarlo, Peggy Möller, Angelika Lander, Larissa Kolesnikova, Stephan Becker

**Affiliations:** 1 Philipps-Universität Marburg, Institut für Virologie, Hans Meerwein-Str. 2, 35032 Marburg, Germany; 2 Promega GmbH, High-Tech-Park, Schildkrötstraße 15, D-68199 Mannheim, Germany; 3 Paul Ehrlich-Institut, Paul-Ehrlich-Str. 51 – 59, 63225 Langen, Germany; 4 Robert Koch-Institut, Zentrum für Biologische Sicherheit, Berlin, Nordufer 20, 13353 Berlin, Germany

## Abstract

The nucleoprotein (NP) of Marburg virus (MARV) is responsible for the encapsidation of viral genomic RNA and the formation of the helical nucleocapsid precursors that accumulate in intracellular inclusions in infected cells. To form the large helical MARV nucleocapsid, NP needs to interact with itself and the viral proteins VP30, VP35 and L, which are also part of the MARV nucleocapsid. In the present study, a conserved coiled coil motif in the central part of MARV NP was shown to be an important element for the interactions of NP with itself and VP35, the viral polymerase cofactor. Additionally, the coiled coil motif was essential for the formation of NP-induced intracellular inclusions and for the function of NP in the process of transcription and replication of viral RNA in a minigenome system. Transfer of the coiled coil motif to a reporter protein was sufficient to mediate interaction of the constructed fusion protein with the N-terminus of NP. The coiled coil motif is bipartite, constituted by two coiled coils which are separated by a flexible linker.

## Introduction

Marburg virus (MARV) and the closely related Ebola virus together make up the family of the *Filoviridae*, which is classified in the order *Mononegavirales*. MARV causes a fulminant hemorrhagic fever in humans and nonhuman primates with high fatality rates [1]. To date, neither a vaccine nor a curative treatment for MARV infection of humans is available. However, live attenuated recombinant vaccines have been described which protected nonhuman primates against MARV and EBOV infections [2,3]. These represent promising candidate vaccines for human use. The recent outbreaks of MARV disease in Angola and Uganda underline the emerging potential of this pathogen [4,5].

The MARV particle is composed of seven structural proteins. Four of them, NP, VP35, VP30 and L, form the nucleocapsid complex of MARV, which surrounds the viral genome [6]. NP, the major nucleocapsid protein, self-assembles into tubular nucleocapsid-like structures, which are mainly found in large intracellular inclusions [7-9]. Formation of the NP tubular structures is presumed to be the first step in nucleocapsid assembly. NP interacts with VP35 which, in turn, interacts with the RNA-dependent RNA polymerase L [6,10]. The complex of VP35 and L acts as the active RNA-dependent RNA polymerase with VP35 serving as polymerase cofactor [11]. Additionally, a trimeric complex was observed consisting of NP, VP35, and L with VP35 connecting L and NP [6]. Three of the four nucleocapsid proteins, NP, VP35, and L, are essential for transcription and replication of the viral RNA [12]. The fourth nucleocapsid protein, VP30, plays an important role in viral transcription of the closely related Ebola virus [11]. For MARV, the role of VP30 is not completely understood at this time. While a minigenome-based transcription/replication system did not indicate a requirement for VP30 in transcription [12], RNAi-based down-regulation of VP30 expression in MARV infected cells resulted in decreased levels of all other viral proteins. This suggests a role for VP30 in replication or transcription.

The self-interaction of NP is the basis for the formation of the helical nucleocapsid of MARV. Most likely, more than one homooligomerization domain is necessary to build the large helices composed of several hundred NP molecules. Additional binding sites on NP mediate interactions with VP35 and VP30. Mapping of the different interaction domains on NP is necessary to understand the different functions of NP during transcription, replication and viral morphogenesis.

In the present study we show that a predicted coiled coil motif in NP is critical for the homooligomerization of NP, formation of NP-induced intracellular inclusions, interaction of NP with VP35 and for the function of NP in RNA synthesis.

## Materials and methods

### Cells and cDNA transfections

HeLa, HUH7 and HUH-T7 cells [13] were grown in Dulbecco's minimal essential medium (Gibco) supplemented with 10% fetal calf serum, 1% glutamine, and 1% antibiotics. Plasmids encoding mutant or wild type MARV proteins were transfected with FuGENE (Roche, Lewes, East Sussex, U.K.) according to the supplier's protocol. The minigenome system was set up according to Mühlberger et al., 1999 [11] with the exception that HUH-T7 cells were used to constitutively express T7 polymerase instead of using HeLa cells and infection with MVA-T7.

### Plasmids

#### Internal deletion mutants of NP (accession number: Z12132)

Deletions of the coiled coil motifs (coiled coil 1: aa 320–348, and coiled coil 2: aa 371–400, coiled coil 1 + 2: aa 320–400) were generated within NP by inverse PCR (Imai et al., 1991) and pT-NP as template [6]. Plasmids containing the required mutation were verified by automated DNA sequencing.

Plasmids encoding NP with sequential deletions of 10 amino acids covering the region 351 – 480 were also generated by inverse PCR.

#### C-terminal deletion mutants of NP

Plasmids encoding C-terminal truncated mutants of NP were generated by insertion of stop codons at the desired position using the site-direted mutagenesis (Stratagene)

#### pTM1-C1C2-M-Flag

Sequence encoding the coiled coil regions (residues 321–400) was amplified by PCR using the plasmid pT-NP. PCR products were cloned into EcoRI and BamHI restriction site of the plasmid pTM1-E30m, which encodes an oligomerization-negative Ebola virus VP30 [14]. The sequence encoding the coiled coil motif was inserted at the 5'-end of the VP30 gene.

#### Bacterial expression vectors

Coding sequences of MARV NP and MARV VP35 genes (accession number: Z12132) were amplified by PCR from pT-NP and pT-VP35, and cloned into the bacterial expression vector pGEX-5x-1 (General Electrics, Freiburg, Germany), respectively, using EcoRI restriction site to generate pGex-NP and pGex-VP35. Sequence of the plasmids was confirmed by automated DNA sequencing.

Detailed descriptions of cloning strategies are available on request.

### Glutathione S tranferase (GST) pull-down assay

pGex-NP and pGex-VP35 were transformed into the BL21 strain of E. coli, and expression of the respective proteins was induced by isopropyl-ß-D-thiogalactopyranoside (IPTG) at a final concentration of 0.2 mM at 2 h after inoculation of the bacteria. For background control, the vector pGEX-5x-1 was transformed in parallel. After 4–5 h of incubation at 30°C, bacteria were harvested by centrifugation and washed twice with PBS containing protease inhibitor cocktail complete™ (2 tablets/ml, Roche, Lewes, East Sussex, U.K.) and Na-orthovanadate (100 μM). The final cell pellet was three times frozen/thawed, incubated on ice for 30 min in buffer 1 (0.5% NP40, 50 mM HEPES, 10% Glycerol, 200 mM NaCl, 0.1% BSA), sonicated (3× 10s at -10°C), incubated for 1 h at 4°C after the addition of 0.1% Triton ×-100. The suspension was cleared by centrifugation (8,000 rpm, 4°C, 10 min). The supernatant was incubated with 50% slurry of GSH sepharose beads (GE-Healthcare, Germany) in presence of 5 mM dithiothreitol (DTT) for 90 min in an overhead rotator at 4°C. Complexes were precipitated, washed twice with ice-cold PBS and once with buffer 1 supplemented with protease inhibitors as described above and 5 mM DTT. The final pellet corresponded to purified GST-NP, GST-VP35, or GST. Radiolabeled in vitro translation products were incubated either with purified GST-NP, GST-VP35, or GST in an overhead rotator for 12 h at 4°C. After two washing steps with buffer 1 and once with buffer 2 (0.5% NP40, 50 mM HEPES, 10% Glycerol, 500 mM NaCl), sepharose beads were resuspended in SDS sample buffer and heated for 5 min at 75°C. All samples were analyzed by 10% SDS PAGE, subsequent Coomassie blue staining (to visualize the bacterially expressed proteins) and quantification of radioactive signals using BioImage analyzer BAS-1000 and the software TINA version 2.0, and Basreader (Raytest, Freiburg, Germany). The amount of input NP bound to GST-NP or to GST-VP35 was set to100%.

### Marburg virus-specific artificial minigenome system

Functional analyses of mutants of NP were performed using a MARV-specific minigenome system essentially as described by Mühlberger et al., [12] with the exception that instead of HeLa cells, HUH-T7 cells were used which expressed the T7 DNA-dependent RNA polymerase. Therefore infection of cells with MVA-T7 was omitted.

### Co-immunoprecipitation, in vitro translation, immunofluorescence analysis

These methods were performed as described by [10].

### MVA-T7-driven expression of proteins

This method was performed as described by Becker et al. [6].

## Results

**Figure 1:**
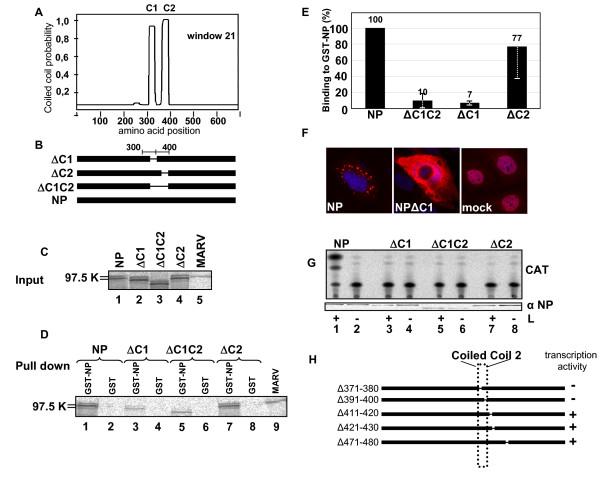
**Role of the coiled coil region in NP for NP self interaction, inclusion body formation and MARV-specific transcription/replication.** (A) In silico analysis of NP predicted two coiled coil motifs at aa position 315 to 400 which are separated by a linker of 23 aa. (B) Schematic presentation of the constructed mutants of NP with deletions in the coiled coil region. (C) The constructed plasmids were in vitro translated, metabolically labeled, separated by SDS-PAGE and analyzed using a BioImager. (D) In vitro translated and metabolically labeled mutants of NP were incubated with bacterially expressed GST-NP or GST (negative control). Complexes were pulled down with glutathion-sepharose, separated on SDS-PAGE and analyzed using a BioImager. Binding of NP to GST-NP was set to 100%. (E) Quantification of 3 separate experiments as shown under (D). (F) Intracellular distribution of NP and NPΔC1. HUH7 cells were transfected with plasmids encoding NP or NPΔC1 together with a plasmid encoding T7 polymerase. Cells were fixed with 4% paraformaldehyde at 18 h post transfection and incubated with a rabbit anti-NP antiserum. Bound antibodies were detected with a rhodamine-coupled donkey anti-rabbit antibody (NP), and a FITC-coupled donkey anti-rabbit antibody (NPΔC1). (G) Impact of coiled coils on the function of NP in a MARV-specific minigenome transcription/replication system. MARV nucleocapsid proteins were expressed in HUHT7 cells together with a MARV specific minigenome. NP was replaced by the indicated mutants of NP and reporter gene activity (CAT) was measured. Below the CAT assay, expression of NP and the NP mutants was confirmed in Western Blot analysis. +: presence of L. -: absence of L (negative control). (H) Results of a transcription/replication analysis using different internal deletion mutants of NP in a minigenome-based assay (G). +: minigenome system active (transcription and replication monitored by CAT assay). -: minigenome system inactive.

A prerequisite for the formation of filoviral nucleocapsids is the homooligomerization of NP, which self-assembles into helical tubules, which are 17 nm in diameter, observed both in cells expressing recombinant NP and in MARV infected cells [8,9]. The formation of the large NP-induced tubules, which are composed of several hundred NP molecules, most likely requires several homooligomerization domains on NP mediating the helical arrangement and the accumulation of the individual tubules into inclusion bodies. In silico analyses of NP (accession number: Z12132) revealed two stretches of 27 aa in the central part of NP (aa 315 to 400) with a high probability to form coiled coils (Fig. 1A, [15]). The two coiled coil motifs are separated by a spacer of 23 aa. Since coiled coils represent a common protein-protein interaction module, we analyzed whether deletion of the individual coiled coil motifs altered the ability of NP to homooligomerize [16,17]. To this end, sequences encoding either the first (Fig. 1B; ΔC1, aa 320–348), the second (ΔC2, aa 371–400), or both coiled coils (ΔC1C2, aa 320 – 400) were removed from the NP-encoding plasmid pT-NP. The resulting mutants were in vitro translated (Fig. 1C) and were subsequently employed in a GST pull-down assay using NP fused to GST (GST-NP). We found that removal of coiled coil 1 (ΔC1) had a significant impact on the binding of NP to itself (Figs. 1D and 1E, ΔC1). The binding strength was also decreased when both coiled coil motifs were deleted (Figs. 1D and 1E, ΔC1C2). Deletion of C2 did not significantly impair the homooligomerization of NP (Figs. 1D and 1E, ΔC2), suggesting that C1, but not C2 is essential for intermolecular interaction between NP molecules.

In MARV infected cells and upon recombinant NP expression, intracellular inclusion bodies are formed that contain accumulated NP-helices [8]. It was of interest to determine whether the deletion of C1, which inhibited NP-NP assembly, influenced the capacity of NP to form inclusion bodies. NP and NPΔC1 were expressed in HUH7 cells which were subsequently subjected to immunofluorescence analysis. While NP expression induced perinuclear inclusion bodies, expression of NP lacking C1 (NPΔC1) resulted in homogenous distribution of NP throughout the cells suggesting that C1 is essential for accumulation of NP-helices into intracellular inclusions (Fig. 1F).

We next tested whether deletion of the coiled coils interfered with the function of NP in transcription and replication of the viral RNA [12]. An artificial MARV-specific transcription/replication system was set up using a CAT gene-containing MARV-specific minigenome as template [12]. In this system, NP was replaced by the different coiled coil mutants and virus-specific transcription was monitored by CAT activity. In the presence of NP, replication and transcription of the minigenome system is active (Fig. 1G, lane 1). Replacement of NP by one of the three coiled coil mutants abolished virus-specific transcription (Fig. 1G, lanes 3, 5, 7). This result indicated that deleting either one or both coiled coil motifs unequivocally abolished the ability of the protein to support viral transcription. In a second approach, smaller deletions were introduced in the region around and inside C2 and the resulting mutants were tested in the artificial minigenome system. Two deletions inside C2 were lethal for the function of the NP (Fig. 1H, Δ371–380, Δ391–400), whereas mutations downstream of the C2 region did not influence the function of NP (Fig. 1G). Together, these results indicated that the coiled coil motifs in NP are important structural elements that support homooligomerization and the functions of the protein.

**Figure 2:**
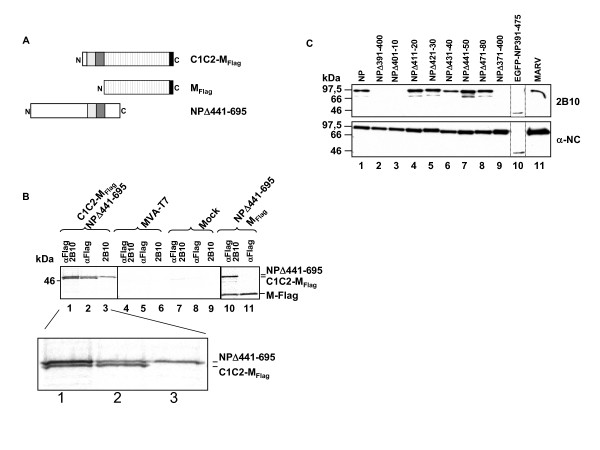
The coiled coil region in NP is sufficient to mediate interaction with NP. (A) Schematic presentation of the constructed mutants. The coiled coil region (C1C2, grey boxes) was fused to an unrelated reporter protein (M_Flag_, striped box). (B) The mutants were expressed using the MVA-T7 system in HeLa cells and metabolically labeled using [^35^S]ProMix. Cells were lysed at 24 h post transfection and cell lysates precipitated using mouse monoclonal anti-Flag (M2) and/or mouse monoclonal anti NP (2B10) as indicated. Precipitates were separated by SDS-PAGE and analyzed using BioImager. (C) Epitope of the mouse monoclonal antibody 2B10 on NP. Mutants of NP with sequential deletions of 10 aa were expressed in HUHT7 cells and cells were lysed at 24 h post transfection. Cell lysates were separated by SDS-PAGE and gels blotted onto polyvinylidene fluoride membrane. Membrane was subjected to Western Blot analysis using either the anti MARV NP mouse monoclonal antibody 2B10 (2B10) or a rabbit anti MARV nucleocapsid antiserum (α-NC).

Next, we aimed to determine whether the coiled motifs are sufficient to mediate protein-protein interactions. The region of the NP gene encoding amino acids 310 to 400 was cloned in frame with a mutant of Ebola virus VP30 (M_Flag_; Fig. 2A, [14]). The fusion protein, named C1C2-M_Flag_, was coexpressed with the N-terminus of NP (NPΔ441–695), which contains the two coiled coil motifs (Fig. 2a, Fig. 2B, lane 1). C1C2-M_Flag _was then specifically precipitated using an anti-Flag antibody; this antibody did not precipitate the NP N-terminus (Fig. 2B lanes 2 and 11). The precipitation anti-Flag antibody resulted in the cosedimentation of the NP N-terminus, suggesting that both proteins were able to interact with each other. However, when a mouse monoclonal anti-NP antibody (2B10) was employed in the coimmunoprecipitation, only NPΔ441–695 was precipitated, suggesting that this antibody inhibited the interaction of the two proteins (Fig. 2B, lane 3). Control experiments showed that the Flag-tagged mutant of VP30 (M_Flag_) was unable to interact with the NPΔ441–695 (Fig. 2B, lanes 10 and 11).

The binding site of the monoclonal antibody 2B10 on NP was analyzed by Western Blot using internal deletion mutants of NP. From the set of tested NP mutants, the ones lacking amino acids 391 – 400 and 401 – 410 were not recognized by 2B10 (Fig. 2C, lanes 2 and 3). Conversely, 2B10 recognized a fusion protein containing the amino acids 391 – 475 fused to EGFP (Fig. 2C, lane 10). These data indicate that the monoclonal antibody 2B10 epitope is located near the coiled coil motifs. We suggest that binding of the monoclonal antibody 2B10 inhibited the interaction between the coiled coil motif and the NP N-terminus by steric hindrance. Taken together, the presented results suggest that the coiled coil motifs in NP are necessary and sufficient to mediate protein-protein interaction.

**Figure 3:**
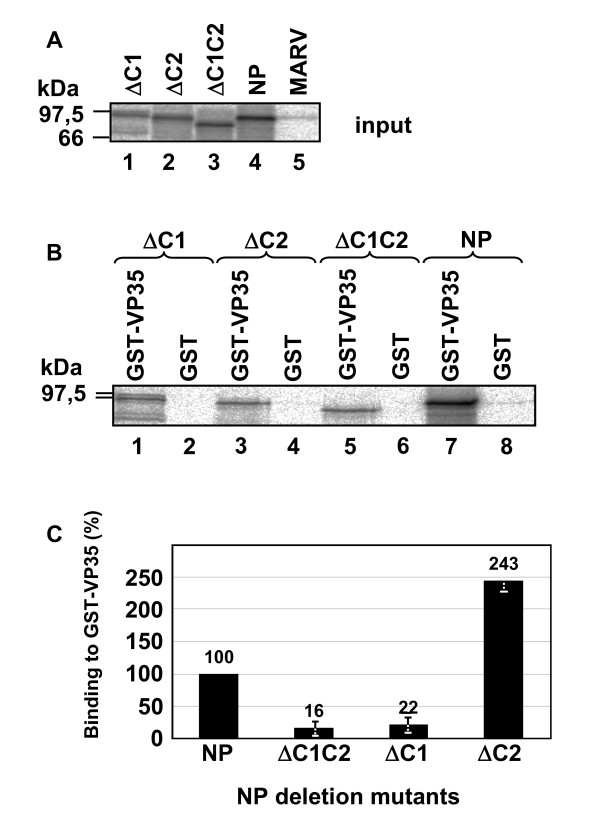
Role of the coiled coil region for interaction of NP with VP35. (A) Mutants of NP (Fig. 1) were in vitro translated and metabolically labeled with [^35^S]ProMix. Samples were separated by SDS-PAGE and analyzed using a BioImager. (B) In vitro translated and [^35^S]ProMix metabolically labeled mutants of NP (Fig. 1A) were incubated with bacterially expressed GST-VP35 or GST (negative control). Complexes were pulled down with glutathion-sepharose, separated on SDS-PAGE and analyzed using a BioImager. Binding of NP to GST-VP35 was set to 100%. (C) Quantification of 3 separate experiments as shown under (B).

We then investigated whether the integrity of the coiled coil region of NP is important for the binding of NP to the polymerase cofactor VP35, which connects the polymerase L to the NP-induced helical nucleocapsid [6,10]. The coding region of VP35 was fused to GST and the fusion protein (GST-VP35) was expressed in E. Coli and, following purification, was incubated with in vitro translated NP or NP mutants containing deletions of the coiled coil motifs (Fig. 3A). In a GST pull-down assay, GST-VP35 was pulled down by glutathione-sepharose and the amount of coprecipitated NP and mutants of NP was quantified to assess the interaction of VP35 with the coiled coils (Figs. 3B and 3C). While NP was readily precipitated by GST-VP35, deletion of C1 significantly decreased the amount of coprecipitated protein (Figs. 3B and 3C, ΔC1). Deletion of C2 seemed to increase the binding of GST-VP35 to the NP mutant (Figs 3B and 3C, ΔC2). These data indicate that the coiled coil region of NP influences the interaction with VP35.

**Figure 4:**
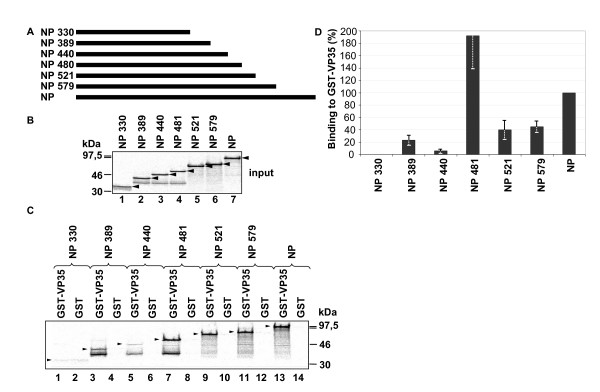
Mapping of regions on NP involved in binding to VP35. (A) Schematic presentation of the constructed mutants of NP with C-terminal truncations. (B) The constructed plasmids were in vitro translated, metabolically labeled using [^35^S]ProMix, separated by SDS-PAGE and analyzed using a BioImager. (C) In vitro translated and metabolically labeled mutants of NP were incubated with bacterially expressed GST-VP35 or GST (negative control). Complexes were pulled down with glutathion-sepharose, separated on SDS PAGE and analyzed using a BioImager. Binding of NP to GST-VP35 was set to 100%. (D) Quantification of 3 separate experiments as shown under (C).

To address the question of whether the interaction domain for VP35 is present in the coiled coil region itself or whether the coiled coil region is necessary to support the integrity of the VP35 binding site, we analyzed the interaction between VP35 and C-terminally truncated NP mutants (Fig. 4A). NP mutants were in vitro translated (Fig. 4B) and incubated with the bacterially expressed fusion protein of VP35 and GST. The resultant complexes were precipitated with glutathione-sepharose and the amounts of precipitated NP mutants were quantified by BioImaging (Figs. 4C and 4D). NP mutants containing the N-terminal amino acids 1–330, 1–389 and 1–440 showed only weak binding to GST-VP35 (Fig. 4C. lanes 1, 3, 5, 7 and Fig. 4D). The inclusion of the next 40 amino acids, which resulted in NP mutant 1–480, increased binding to GST-VP35 significantly (Fig. 4C, lane 9 and 3D). Further elongation of the protein, however, diminished binding of the respective NP mutant to GST-VP35. These results suggested that the interaction between NP and VP35 is dependent on the coiled coil region and amino acids 440 to 480.

Taken together, the coiled coil region of NP is essential and sufficient to mediate the interactions between NP molecules and is necessary for its interaction with VP35. Moreover, the presence of the coiled coil domains is essential for the function of NP in RNA synthesis.

## Discussion

Coiled coil motifs are versatile domains that mediate the interaction of proteins [16]. The central feature of coiled coil motifs is the presence of repeats of a heptameric amino acid sequence (heptad repeats, a – to – g) with hydrophobic residues at the key positions, a and d. The coiled coils fold into condensed helical structures that are able to interact, via the amino acids at position a and d, with another coiled coil in a "knob-into-holes" manner. This arrangement results in a stable hydrophobic interaction between two coiled coil motifs. Coiled coil motifs are able to form intra- and intermolecular bonds [18].

**Figure 5:**
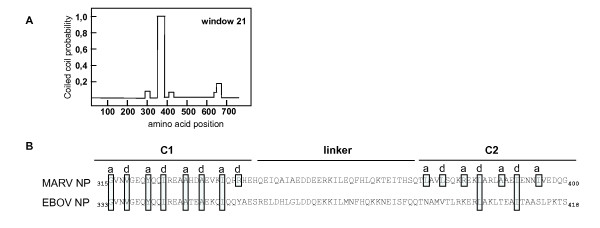
Coiled coil motif in Zaire Ebola virus NP (A) In silico analysis of EBOV NP predicted one coiled coil motifs at aa position 333 to 367. (B) Alignment of the coiled coil regions in NP of filoviruses. C1: coiled coil motif 1, C2: coiled coil motif 2. Linker: Sequence without coiled coil prediction. a, d: Key positions in the heptad repeats of MARV NP. Large boxes: conserved amino acids at the coiled coil key positions. Small boxes: Key positions in MARV NP without conservation in Ebola virus NP.

We have recently reported that homooligomerization of VP35, the polymerase cofactor, is mediated by a coiled coil motif which leads to the formation of tetramers [10]. Here we show that the predicted coiled coil domains in NP, the major MARV nucleocapsid protein, play an essential role in the formation of NP-NP oligomers and the formation of inclusion bodies which contain preformed NP-induced nucleocapsid-like structures [8]. The nucleoprotein of Ebola virus also contains a region, encompassing amino acids 334 to 363, which has a very high probability to form a coiled coil structure (Fig. 5A). The predicted coiled coil in Ebola virus NP corresponds to the predicted coiled coil C1 of MARV NP. Seven of the nine key residues of C1 are conserved between MARV and ZEBOV (Fig. 5B). Interestingly, the second coiled coil predicted in MARV NP (amino acid positions 371 – 400) is less conserved in Ebola virus NP (Fig. 5B).

The presence of C1 is essential for the formation of the NP-NP and the NP-VP35 complex, while removal of C2 has only a mild inhibitory effect in the case of NP-NP complex formation and it even enhances binding in the case of the NP-VP35 interaction. On the other hand, C2 is essential for the function of NP in transcription/replication. Sequential 10 amino acid deletions both inside and outside of coiled coil motif C2 underline this result by revealing that only deletions inside C2 abolished the function of NP (Fig. 1H). These results support the following hypothesis. The two coiled coil motifs are involved in intra- and intermolecular binding. While C1 is involved in intermolecular binding between NP molecules and supports binding of NP and VP35, the second coiled coil motif (C2) mediates an intramolecular interaction with C1. The intramolecular interaction might be involved in regulating binding between NP and VP35 (binding is enhanced in the absence of C2) and is essential for the function of NP in transcription and replication of MARV RNA.

It is possible that the conformational flexibility of NP, which allows for both intra- and intermolecular binding, is a prerequisite for NP to perform its multiple tasks in RNA synthesis and nucleocapsid morphogenesis. This concept is supported by characterization of the Hantavirus NP. Alfadhli et al. showed that the predicted coiled coil in Hantavirus NP facilitates intramolecular binding via a helix turn helix structure at low concentrations, while it facilitates intermolecular binding at high concentrations [18]. Additionally, the 3D structure of the vesicular stomatitis virus nucleocapsid protein complexed to RNA suggests that the conformation of the nucleocapsid protein must undergo changes to allow the polymerase complex access to the RNA [19].

The formation of complex helical structures composed of hundreds of proteins is only possible if several homotypic interaction domains are available that allow the sequential ordered arrangement of the molecules. Homooligomerization of NP via the coiled coil motif in a central region of NP does not exclude the presence of other homooligomerization domains in the protein. To that end, Watanabe et al. presented data for Ebola virus showing that the presence of the C-terminal 150 amino acids of NP is necessary for the formation of the helical nucleocapsids [20]. It might be that the coiled coil-mediated binding of NP molecules to each other is only one step in the formation of the helix which is then followed by or accompanied by interactions with other parts of NP.

For other viruses of the order Mononegavirales, Sendai virus and measles virus, a conserved central part of NP has been found to be necessary for homooligomerization of NP [21-23]. Interestingly, the respective regions in NP or N proteins do not have a high probability of forming coiled coil structures.

In this study, we have shown that two predicted coiled coil motifs in NP of MARV are important structural elements for NP-NP and NP-VP35 interactions and the formation of inclusions induced by NP. The coiled coil motifs can be transferred to an unrelated reporter protein and are sufficient to mediate the interaction between the reporter protein and NP. Moreover, both motifs are essential for the function of NP during transcription and replication.
